# Specifying and comparing implementation strategies across seven large implementation interventions: a practical application of theory

**DOI:** 10.1186/s13012-019-0876-4

**Published:** 2019-03-21

**Authors:** Cynthia K. Perry, Laura J. Damschroder, Jennifer R. Hemler, Tanisha T. Woodson, Sarah S. Ono, Deborah J. Cohen

**Affiliations:** 1 School of Nursing, Oregon Health & Science University, 3455 SW US Veterans Hospital Rd, Portland, OR 97239 USA; 2 Implementation Pathways, LLC, Ann Arbor, MI USA; 3 VA Center for Clinical Management Research, Ann Arbor, MI USA; 4 Department of Family Medicine and Community Health, Rutgers University--Robert Wood Johnson Medical School, 112 Paterson Street, New Brunswick, NJ 08901 USA; 5 Department of Family Medicine, Oregon Health & Science University, 3181 SW Sam Jackson Park Rd, Portland, OR 97239 USA

**Keywords:** Implementation strategy mapping, Implementation strategies, Large-scale initiative, Capacity building, Implementation facilitation

## Abstract

**Background:**

The use of implementation strategies is an active and purposive approach to translate research findings into routine clinical care. The Expert Recommendations for Implementing Change (ERIC) identified and defined discrete implementation strategies, and Proctor and colleagues have made recommendations for specifying operationalization of each strategy. We use empirical data to test how the ERIC taxonomy applies to a large dissemination and implementation initiative aimed at taking cardiac prevention to scale in primary care practice.

**Methods:**

EvidenceNOW is an Agency for Healthcare Research and Quality initiative that funded seven cooperatives across seven regions in the USA. Cooperatives implemented multi-component interventions to improve heart health and build quality improvement capacity, and used a range of implementation strategies to foster practice change. We used ERIC to identify cooperatives’ implementation strategies and specified the actor, action, target, dose, temporality, justification, and expected outcome for each. We mapped and compiled a matrix of the specified ERIC strategies across the cooperatives, and used consensus to resolve mapping differences. We then grouped implementation strategies by outcomes and justifications, which led to insights regarding the use of and linkages between ERIC strategies in real-world scale-up efforts.

**Results:**

Thirty-three ERIC strategies were used by cooperatives. We identified a range of revisions to the ERIC taxonomy to improve the practical application of these strategies. These proposed changes include revisions to four strategy names and 12 definitions. We suggest adding three new strategies because they encapsulate distinct actions that were not described in the existing ERIC taxonomy. In addition, we organized ERIC implementation strategies into four functional groupings based on the way we observed them being applied in practice. These groupings show how ERIC strategies are, out of necessity, interconnected, to achieve the work involved in rapidly taking evidence to scale.

**Conclusions:**

Findings of our work suggest revisions to the ERIC implementation strategies to reflect their utilization in real-work dissemination and implementation efforts. The functional groupings of the ERIC implementation strategies that emerged from on-the-ground implementers will help guide others in choosing among and linking multiple implementation strategies when planning small- and large-scale implementation efforts.

**Trial registration:**

Registered as Observational Study at www.clinicaltrials.gov (NCT02560428).

**Electronic supplementary material:**

The online version of this article (10.1186/s13012-019-0876-4) contains supplementary material, which is available to authorized users.

## Background

It is well recognized that an active and purposive approach is required to translate research findings into routine clinical care. Methods used to disseminate and implement research findings into clinical practice are termed implementation strategies, [1] and interventions designed to facilitate the application of evidence into clinical practice typically use a combination of implementation strategies tailored to a particular clinical context. Multiple implementation frameworks [2, 3] provide guidance on how to define factors that may explain or predict implementation outcomes and how to assess implementation interventions (e.g., Consolidated Framework for Implementation Research (CFIR) [4]; Promoting Action on Research in Health Services (PARIHS) [5]; Normalization Process Theory (NPT) [6]). These frameworks provide theoretical structures and are an indispensable foundation for developing testable hypothesized models, establishing an understanding of the factors that impact implementation, and building a base of implementation knowledge [7, 8]. However, these frameworks may not necessarily provide guidance about how to organize detailed implementation strategies to achieve specific outcomes, may use inconsistent names for implementation strategies, and may lack adequate strategy descriptions—making understanding and replication difficult.

Powell and colleagues sought to address the problem of multiple names and definitions for implementation strategies in 2012 [9]. Through a comprehensive review of the literature, they identified 68 discrete strategies and organized them into six key implementation domains. These domains were organized by type, with similar implementation strategies grouped into the same domain. They then updated and expanded this list by engaging a broad group of implementation scientists in a three-phase, modified-Delphi process that refined and expanded the list to 73 discrete implementation strategies, each with a name and definition [10]. These 73 strategies were grouped into nine clusters based upon strategy type, and ratings of importance (high/low) and feasibility (high/low) were elicited for each strategy [11]. This updated compilation is known as the Expert Recommendations for Implementing Change (ERIC).

ERIC has made an important theoretical contribution to identifying a broad range of implementation strategies and developing a common nomenclature with definitions. An important next step toward advancing this work is to test and refine the taxonomy based on the use of these implementation strategies in on-the-ground implementation efforts; Proctor and colleagues [1] recognized this need and have started this work. They have recommended that implementation strategies be further specified to include such details as the actor (who is enacting the implementation strategy), the actions (specific activities to support implementation), the targets (entity impacted), temporality, dose, expected outcome, and justification. Researchers have used the ERIC and Proctor specifications to understand the operationalization of implementation strategies and have found this useful [12–16]. The level of specification has been helpful in identifying how strategies can be tailored to different settings [14], fostering comparison across clinical sites in a multisite study [16], and helping to inform the development of activity logs to track implementation strategies [13].

Researchers who applied the ERIC and Proctor frameworks to real-world implementation efforts recommended additional implementation strategies for the ERIC taxonomy [13]. Others have used ERIC to guide detailed mapping of implementation strategies during implementation study planning [12]; one of these studies used ERIC to develop a blueprint to guide implementation efforts [15]. As all implementers know, however, there is a big difference between what you propose or plan to do during implementation and what really happens. To date, few studies (and none we could identify) use data from large-scale, multi-site implementation efforts to apply and refine the ERIC taxonomy.

This paper reports a study we conducted to apply the ERIC taxonomy and the Proctor reporting recommendations to multiple large-scale studies. We use empirical data collected during active implementation with an intent of further refining ERIC, if changes were identified as necessary.

## Methods

### Setting

The setting for this study is the EvidenceNOW initiative [17] funded by the Agency for Healthcare Research and Quality (AHRQ). EvidenceNOW focused on rapid dissemination and implementation of cardiovascular preventive care (appropriate aspirin use, blood pressure and cholesterol management, and smoking cessation—the ABCS of heart health) among smaller primary care practices as well as capacity building (e.g., quality improvement experience, data access, practice leadership). In the request for applications, AHRQ “strongly encouraged” the use of specific implementation strategies, including practice facilitation, data review and feedback and benchmarking, peer-to-peer learning, and expert consultation [18], and each cooperative was required to produce clinical quality measures and practice capacity measurement to determine change over time in these domains. In addition to funding seven cooperatives, AHRQ also funded a national evaluation of EvidenceNOW called Evaluating System Change to Advance Learning and Take Evidence to Scale (ESCALATES) to harmonize and coordinate the collection of quantitative data, to collect additional qualitative data, and to bring together the cross-cooperative comparative findings. For more details on the ESCALATES evaluation, see Cohen et al. [19].

### Sample

EvidenceNOW funded seven regional cooperatives that spanned 12 states (Oregon, Washington, Idaho, Colorado, New Mexico, Oklahoma, Wisconsin, Illinois, Indiana, New York City, North Carolina, Virginia). For details on the regions and states, see the EvidenceNOW website [17] and Ono et al. [20]. Each cooperative engaged over 200 primary care practices in this effort (*N* = 1721). Practices were smaller (< 10 clinicians) and varied with respect to ownership and geographic location. Accomplishing the goal of large-scale rapid dissemination and implementation in less than 3 years required a large workforce and use of a range of implementation strategies.

### Data sources

We reviewed and triangulated four sources of qualitative data from years one and two of the EvidenceNOW initiative to identify and define the cooperatives’ implementation strategies. First, we reviewed the cooperatives’ study proposals, which provided an outline of broad, overarching implementation strategies. Second, we examined cooperative-level documents, including training tools and conceptual models, to further specify content of these overarching strategies. We also analyzed facilitator training documents, which provided insights into specific activities and implementation strategies that facilitators would use to support practices. For example, templates for facilitators to document and track their work provided detailed descriptions of activities that facilitators may perform. The third source of data were entries written by cooperative team members on the ESCALATES interactive online diary [21]. The online diary [21] is a web-based platform that allowed our evaluation team to communicate with each cooperative team about their implementation experiences and clarify details about on-the-ground implementation support they were providing to practices. These entries were made during active implementation of the interventions across cooperatives. The fourth data source analyzed comprised field notes from site visits to cooperatives and transcripts from interviews with cooperative leadership and key team members during active implementation. Analyzing interview transcripts and field notes provided important insight into the pragmatic, on-the-ground use of implementation strategies to support practice change.

All field notes were prepared soon after the site visit. Interviews followed a semi-structured guide and were professionally transcribed, checked for accuracy, and de-identified. Field notes, interviews, and other documents were entered into Atlas.ti for data management and analysis.

### Data analysis

We used an iterative and inductive analytic approach [22] to identify and describe cooperatives’ implementation strategies. Guided by Proctor and colleagues’ recommendations for specifying and reporting details on implementation strategies [1], we constructed an intervention table for each cooperative describing the broad implementation strategies and more detailed activities that cooperatives included in their interventions and specified the actors, action targets, dose, temporality, expected outcomes, and justification for each. We member-checked these tables with the cooperative leadership and refined these documents, as needed. (see Additional file 1 for an example of one such table). From these seven intervention tables, we identified 266 detailed actions that were documented by cooperative members to help practices implement changes to improve capacity or ABCS.

Next, four of the authors (LJD, CKP, JRH, TTW) independently mapped each action to a strategy in the ERIC compilation [10] based on alignment of each action with the definition for each ERIC strategy. We met weekly over 5 months to independently map and discuss mappings until we reached consensus. Throughout this process, we operationalized ERIC definitions to help guide decisions about alignment of ERIC implementation strategies with the EvidenceNOW actions and identified areas where ERIC definition expansion, revision, and/or reorganization was needed to better reflect the actions cooperatives used and how that could help make the ERIC strategies more pragmatic and easily applied by researchers and practitioners.

Next, we created a consolidated cross-cooperative matrix that organized implementation strategies based upon their practical application. We pile-sorted each implementation strategy into functionally similar groups based on their justification and expected outcomes. We reviewed and labeled each grouping and considered the cooperatives’ practical applications of strategies within and across the groupings, recognizing interdependencies and differences, such as “actors” (who enacts the strategies within a grouping; facilitators with health information technology (HIT) expertise versus cooperative-employed data experts).

## Results

The 266 actions identified across the seven cooperatives mapped to 33 of the 73 ERIC implementation strategies. We propose refinements for 13 strategies. Table 1 lists the ERIC strategies, our proposed changes, and our rationale for these changes*.* Recommended refinements include changes in strategy names for four strategies and changes in definitions for 12 strategies. Additionally, we propose adding three new distinct strategies which we identified as being used in practices among the cooperatives: *assess and redesign workflow*, *create online learning communities*, and *engage community resources*. Definitions for these strategies are provided in Table 1. The ERIC strategies taxonomy included “Ancillary Material” that the authors included as an Additional file [10]. We recommend changes in ancillary material for 15 strategies and provide ancillary material for the three new strategies; details are provided in our Additional file 2. We will refer to ERIC strategies using our new proposed labels when applicable (e.g., *implementation facilitation* instead of ERIC’s original name of *facilitation*).

**Table 1:** Proposed changes to ERIC strategy labels and/or definitions with rational for changes (proposed changes are in italics)

ERIC name	Current ERIC definition	Proposed changes to definition	Rationale for proposed change
Use data experts	Involve, hire, and/or consult experts to inform management on the use of data generated by implementation efforts	Involve, hire, and/or consult experts to *acquire*, *structure*, *manage*, *report*, *and use* data generated by implementation efforts	We broadened functions of data experts beyond just management of data.
Fund and contract *and/or negotiate with vendors* for the clinical innovation	Governments and other payers of services issue requests for proposals to deliver the innovation, use contracting processes to motivate providers to deliver the clinical innovation, and develop new funding formulas that make it more likely that providers will deliver the innovation	None	We broadened the name to include the role of negotiation. Having outside assistance to negotiate with EHR vendors can be valuable in addition to payment.
Provide local technical assistance	Develop and use a system to deliver technical assistance focused on implementation issues using local personnel	Develop and use a system to deliver technical assistance *within local settings that is* focused on implementation issues	We clarified the definition to indicate any technical assistance provided in the local setting, whether provided by local staff or by other on-site individuals.
Audit and provide feedback	Collect and summarize clinical performance data over a specified time period and give it to clinicians and administrators to monitor, evaluate, and modify provider behavior	*Develop summaries of* clinical performance over a specific time period, *often including a comparator*, and give it to clinicians and/or administrators. *Summary content* (e.g., *nature of the data*, *choice of comparator*) *and their delivery* (e.g., *mode, format*) *are designed* to modify *specifically targeted behavior*(*s*) *or actions of individual practitioners*, *teams*, *or health care organizations*	We broadened the definition to include providing comparator data (benchmark) and to indicate that goal is to modify a targeted behavior and/or action of multiple actors. These facets are listed in ancillary materials but were not included in the published definition.
Use an implementation advisor	Seek guidance from experts in implementation	Seek guidance from experts in implementation, *including providing support and training for the implementation work force*	We broadened this definition to include providing support and training for facilitators.
*Implementation* facilitation	A process of interactive problem solving and support that occurs in a context of a recognized need for improvement and a supportive interpersonal relationship	*[A] multi-faceted* interactiv*e process of* problem solving, *enabling and supporting individuals*, *groups and organizations in their efforts to adopt and incorporate innovations into routine practices* that occurs in a context of a recognized need for improvement and a supportive interpersonal relationship	The name was changed to specify “implementation” because facilitation is a very broad concept. Implementation facilitation includes practice facilitation, a more specific type of implementation facilitation. We broadened the definition to acknowledge that facilitation is more than just “interactive problem-solving.”
Assess for readiness and identify barriers and facilitators	Assess various aspects of an organization to determine its degree of readiness to implement, barriers that may impede implementation, and strengths that can be used in the implementation effort	Assess various aspects of an organization to determine its degree of readiness to implement *and identify* barriers that may impede implementation and strengths that can be *leveraged to facilitate* the implementation effort	We revised to clarify identification of barriers and leveraging facilitators.
Develop *an* implementation blueprint	Develop a formal implementation blueprint that includes all goals and strategies. The blueprint should include the following: (1) aim/purpose of the implementation; (2) scope of the change (e.g., what organizational units are affected); (3) timeframe and milestones; and (4) appropriate performance/progress measures. Use and update this plan to guide the implementation effort over time	Develop a formal implementation blueprint that includes all goals and strategies. The blueprint should include the following: (1) aim/purpose of the implementation; (2) scope of the change (e.g., what organizational units are affected); (3) timeframe and milestones; and (4) appropriate performance/progress measures. *Use and update this plan to guide the implementation effort over time*	We suggest deleting the word “Formal” to include informal as well as formal implementation blueprints. This will include plans that are developed for quality improvement as well as larger formal plans for implementation. Additionally, the definition is expanded to explicitly acknowledge its role in guiding implementation over time.
Organize implementation team*s and team* meetings	Develop and support teams of clinicians who are implementing the innovation and give them protected time to reflect on the implementation effort, share lessons learned, and support one another’s learning	Develop and support teams of clinicians, *staff*, *patients and other stakeholders* who are implementing *or may be users of* the innovation. *Provide* protected time *for teams* to reflect on the implementation *progress*, share lessons learned, *make refinements to plans*, and support one another’s learning	We broadened the name to include all possible team members and to include formation of teams as well as meetings. Removing the term clinician allows for a multi-disciplinary team and increases engagement among all team members. We broadened definition to be inclusive of all team members and clarified intent of the definition.
Develop educational materials	Develop and format manuals, toolkits, and other supporting materials in ways that make it easier for stakeholders to learn about the innovation and for clinicians to learn how to deliver the clinical innovation	Develop and format manuals, toolkits, and other supporting materials *to* make it easier for stakeholders to learn about the innovation and for clinicians to learn how to deliver the clinical innovation*. This can include technology-delivered* (e.g., *online/smartphone-based static or dynamic*) *content and health messaging*	We expanded to include technology-delivered content and messaging.
Conduct educational outreach visits	Have a trained person meet with providers in their practice settings to educate providers about the clinical innovation with the intent of changing the provider’s practice	Have a trained person meet with *individuals or teams* in their *work* settings to educate *them* about the clinical innovation with the intent of changing *behavior to reliably use the clinical innovation as designed*	We broadened definition to include team members beyond providers and clarified language to more clearly state that this strategy aims to encourage sustained use of the innovation.
Conduct ongoing training	Plan for and conduct training in the clinical innovation in an ongoing way	Plan for and conduct training in the clinical innovation in an ongoing way *for all individuals involved with implementation and users of the clinical innovation* e.g., *clinicians*, *implementation staff*, *practice facilitators*	We expanded the definition to include all individuals involved.
Conduct educational meetings	Hold meetings targeted toward different stakeholder groups (e.g., providers, administrators, other organizational stakeholders, and community, patient/consumer, and family stakeholders) to teach them about the clinical innovation	Hold meetings targeted toward *educating multiple* stakeholder groups (i.e., providers, administrator*s*, other organizational stakeholders, community *members*, patient*s*/consumer*s*, famil*ies*) *about the clinical innovation and/or its implementation*	We revised the definition to add specificity about the purpose of the education (the innovation and/or its implementation) and to clarify that education is among multiple types of stakeholders.
*Assess and redesign workflow*		Observe and map current work processes and plan for desired work processes, identifying changes necessary to accommodate, encourage, or incentivize use of the clinical innovation as designed	New: Added as this work is not reflected in current ERIC strategies.
*Create online learning communities*		Create an online portal for clinical staff members to share and access resources, webinars, and FAQs related to the specific evidenced-based intervention, and provide interactive features to encourage learning across settings and teams, e.g., regular blogs, facilitated discussion boards, access to experts, and networking opportunities	New: Added as this work is not reflected in current ERIC strategies.
*Engage community resources*		Connect practices and their patients to community resources outside the practice (e.g., state and county health departments; non-profit organizations; resources related to addressing the social determinants of health; and organizations focused on self-management techniques and support	New: Added as this work is not reflected in current ERIC strategies.

Please see Additional file 2 for complete list of proposed changes plus changes to “Ancillary Material” originally included in Additional File 6 published with Powell et al. (10)

We grouped the 33 ERIC strategies used by the cooperatives into four functional groupings: (1) build health information technology to support data-informed quality improvement (QI), (2) build QI capacity and improve outcomes, (3) enhance clinician and practice member knowledge, and (4) build community connections and patient involvement. Tables 2, 3, 4, and 5 list implementation strategies and identify the ERIC cluster to which each strategy belongs along with the actor, specific actions, and targets; each table covers of one of the four groupings listed above. The following sections describe the four overarching functions and the implementation strategies within each grouping.

**Table 2:** Build health information technology to support data-informed QI. ERIC strategies included in EvidenceNOW interventions with Proctor specifications

Expected outcomes: Ability to generate ABCS reports sufficient HIT capacity, ability to generate ABCS reports, ABCS documentation, data interoperability and sharing. Sustained performance quality measurement and use of data for QI Justification: Practices need population-level data and interoperability to improve quality and optimize care delivery, but they have little or no HIT capacity for this. Practices may need additional, external data infrastructure to provide robust practice-level, clinician-level, and patient-level data on demand. Practices often need internal support to access, validate, and use data for QI. Practices are motivated to improve upon seeing their own data					
No. co-ops	Name it		Specify it		
	ERIC cluster	ERIC strategy*	Actor	Specific action(s)	Target
5	Adapt and tailor to context	Use data experts	Cooperative leadership, data experts	Hire health informatics technology experts (called “data experts” in this table) to connect practices to external data infrastructures or hire practice facilitators skilled in health informatics technology (called HIT-PFs) to assist practices in generating and understanding EHR data	Cooperative, practice
5	Adapt and tailor to context	Use data warehousing techniques	Data expert, HIT-PF	Connect practice EHR to warehouse, repository, and/or other external durable infrastructures (i.e., registries and software platforms)	Practice
6	Use evaluative and iterative strategies	Develop and implement tools for quality monitoring	Data expert, HIT-PF	Perform the data extraction, data normalization, and “back-end” data validation necessary for data warehousing and other data platforms	Practice, data infrastructure
6	Use evaluative and iterative strategies	Develop and organize quality monitoring systems	Data expert, HIT-PF, PF	Connect practices to additional data interfaces for receiving ABCS data and other types of metrics (i.e., access to data software platforms like popHealth or Sharepoint or cooperative dashboards)	Practice
3	Utilize financial strategies	Fund and contract (*and*/*or negotiate*) *with vendors* for the clinical innovation	Cooperative leadership	Reimburse for registry connections for the duration of EvidenceNOW; negotiate with EHR vendors to connect practices to data infrastructure	Practice
2	Change infrastructure	Change records systems	Data expert	Help practices upgrade to new EHR and/or modify existing EHRs for efficiency and accuracy	Practice
6	Provide interactive assistance	Provide local technical assistance	Data expert, HIT-PF, PF	Audit charts to validate data reports; assist with helping practices improve ABCS documentation; help practices run/generate/pull reports; troubleshoot dashboards; help practice staff transition to and learn to use new EHR, if needed	Practice, PF
7	Use evaluative and iterative strategies	Audit and provide feedback	Data expert, HIT-PF, PF	Share ABCS data for feedback and monitor improvement over time. Most used ABCS data for audit and provide feedback, and several provided benchmarking to similar practices; a few included survey items and other sources of data for feedback	Practice

*ABCS* aspirin, blood pressure, cholesterol, and smoking cessation; *PF* practice facilitator; *HIT* health information technology; *HIT-PF* health information technology specialist practice facilitator; *EHR* electronic health record; *Data expert* health informatics technology expert, *expert consultant* physician faculty or clinical expert; *Q & A* question and answer; *FAQ* frequently asked questions; *extension agent* community-based agent specializing in using community resources to address social determinants of health

*Modifications to ERIC labels and new strategies are in italics. Strategy definitions are in Table 1

**Table 3:** Build QI capacity and improve outcomes. ERIC strategies included in EvidenceNOW interventions with Proctor specifications

Expected outcomes: Improve ABCS outcomes and QI capacity Improved ABCS measures and increased practice capacity to take on new quality initiatives. Additional outcomes may vary by cooperative (e.g., joy in practice). Knowledge of evidence-based guidelines and QI techniques and principles is needed Justification: practices are under-resourced for quality improvement and need external support to guide them through the change process. Implementation staff needs a supportive infrastructure to help them help practices achieve their goals					
No. co-ops	Name it		Specify it		
	ERIC cluster	ERIC strategy*	Actor	Specific action(s)	Target
7	Provide interactive assistance	*Implementation* facilitation	PF	Assist with the change process and support change efforts; review workflows and create actionable plans to put best practices in place (i.e., huddles, pre-visit planning, medication synchronization, gaps analysis, outreach, decision support use, patient education, etc.)	Practice
7	Use evaluative and iterative strategies	Assess for readiness and identify barriers and facilitators	Data expert HIT-PF, PF	Assess HIT needs and data set-up and workflow; assess clinic workflows; assess use of evidence-based protocols	Practice
7	Use evaluative and iterative strategies	Develop *an* implementation blueprint	PF, HIT-PF	Discuss and identify improvement plan; adjust plans according to data; develop actionable plans for implementation of evidence-based protocols and addressing other pain points (i.e. non-compliant patients)	Practice
6	Use evaluative and iterative strategies	Conduct cyclical small tests of change	PF	Develop and use Plan Do Study Act (PDSA), Define Measure Analyze Improve and Control (DMIAC), and other tests of change activities and processes involved in QI	Practice
5	New	*Assess and redesign workflow*	PF	Assess and revise clinic workflows using a variety of techniques (i.e., observations and mapping of workflow, role-redesign exercises) to facilitate discussion and implementation of evidence-based protocols	Practice
4	Support clinicians	Remind clinicians	PF	Assist the change process through use of clinical reminders and decision-support tools	Practice
4	Develop stakeholder interrelationships	Identify and prepare champions	PF	Assist in creating and engaging practice leaders and others in promoting efficient care strategies	Practice
4	Develop stakeholder interrelationships	Recruit, designate and train for leadership	PF	Assist in creating and facilitating practice leaders and others in promoting efficient care strategies	Practice
4	Develop stakeholder interrelationships	Organize *implementation teams and team meetings*	PF	Assist in creating and facilitating QI teams in promoting efficient care strategies	Practice
4	Engage consumers	Intervene with patients/consumers to enhance uptake and adherence	PF	Help generate patient lists for outreach from EHRs or registries; recall patients; offer patient education materials	Practice
5	Develop stakeholder interrelations	Use an implementation advisor	Expert consultant PF-HIT	Support and education for practice facilitators by data experts and/or physician faculty hired as expert consultants	PF

*ABCS* aspirin, blood pressure, cholesterol, and smoking cessation; *PF* practice facilitator; *HIT* health information technology; *HIT-PF* health information technology specialist practice facilitator; *EHR* electronic health record; *data expert* health informatics technology expert; *expert consultant* physician faculty or clinical expert; *Q & A* question and answer, *FAQ* frequently asked questions, *extension agent* community-based agent specializing in using community resources to address social determinants of health

*modifications to ERIC labels and new strategies are in italics. Strategy definitions are in Table 1

**Table 4:** Enhance clinician and practice member knowledge. ERIC strategies included in EvidenceNOW interventions with Proctor specifications

Expected outcomes: Clinician knowledge of ABCS & QI and exchange best practices Knowledge of clinical guidelines, use of local resources, and increased peer interaction encouraging continued engagement and participation in QI. Exchange of best practices, peer learning and support Justification: Clinicians benefit from learning in a variety of formats (peer-to-peer; online learning; in-person one-on-one or group learning) from different professionals (expert consultants who are also clinical peers, practice facilitators, other healthcare experts). Interactive, collaborative learning and peer networking help practices engage in QI and reduce burnout					
No. co-ops	Name it		Specify it		
	ERIC cluster	ERIC strategy*	Actor	Specific action(s)	Target
7	Train and educate stakeholders	Develop educational materials	Cooperative leadership, expert consultants, PF	Develop webinars, Q & A, toolkit, training modules, and online resources; compile lists of community resources	Practice
7	Train and educate stakeholders	Distribute educational materials	Expert consultant, community health improvement organization, extension agent, PF	Host didactic webinars and videos and post toolkits, modules, Q & A/FAQ online for asynchronous learning; provide information during in person visits, such as listing of community resources; support change efforts through providing educational materials and working on them with practices; establish links to community resources	Practice
1	Train and educate stakeholders	Conduct educational outreach visits	Expert consultants, data experts	Hold one-on-one meetings with practice to educate on clinical topics; visits to train practice data champion in data reporting/data use training	Practice
3	Train and educate stakeholders	Provide o*ngoing* consultation	Expert consultants	Provide expert advice by email or in person	Practice
3	Train and educate stakeholders	Conduct *ongoing* training	Expert consultants	Provide interactive training webinars and “office hours” on clinical topics related to the ABCS; include time for Q & A. Provide training on cardiovascular disease risk calculators and other clinical tools	Practice
3	Train and educate stakeholders	Create a learning collaborative	Cooperative leadership, PF, practice	Hold events for practices to learn from and interact with cooperative staff about the clinical interventions as well as interact with other practices, workshop practice needs, and create plans for embarking on or continuing with intervention change processes	Practice
2	New	*Create online learning communities*	Cooperative leadership, PF, practice	Create online forum with access to resources, networking, and discussions	Clinical community
5	Train and educate stakeholders	Conduct educational meetings	Cooperative leadership, expert consultants, PF, practice	Hold collective meetings for practices with cooperatives for the purpose of orienting the practice to the intervention and educating practice members on clinical topics and/or best practices; practice facilitators may hold collaborative calls or meetings with practices for training on particular topics of practices’ choice	Practice
1	Train and educate stakeholders	Shadow other experts	Cooperative leadership, practice	Select exemplar practices for other practices to visit; visits may be in person or virtual	Practice
2	Develop stakeholder interrelations	Visit other sites	Cooperative leadership, practice	Select exemplar practices for other practices to visit or bring together local practices to discuss best practices and intervention successes and challenges; visits may be in person or virtual	Practice
4	Develop stakeholder interrelations	Promote network weaving	Cooperative leadership, PF, practice	Encourage networking between practices to build a clinical community for best practices	Practice
5	Develop stakeholder interrelation-ships	Capture and share local knowledge	Cooperative leadership, expert consultants, PF, practice	Hold in-person meetings or events or phone calls so that practices can learn from each other and engage in discussion; visit exemplar practices; facilitate networks and learning events in the region; host a small group of practices in one clinic; use online portal for virtual learning	Practice

*ABCS* aspirin, blood pressure, cholesterol, and smoking cessation; *PF* practice facilitator; *HIT* health information technology; *HIT-PF* health information technology specialist practice facilitator; *EHR* electronic health record; *data expert* health informatics technology expert; *expert consultant* physician faculty or clinical expert; *Q & A* question and answer; *FAQ* frequently asked questions; *extension agent* community-based agent specializing in using community resources to address social determinants of health

*Modifications to ERIC labels and new strategies are in italics. Strategy definitions are in Table 1

**Table 5:** Build community connections and patient involvement. ERIC strategies included in EvidenceNOW interventions with Proctor specifications

Expected outcomes: Improved community connections and patient involvement Improved referrals to accessible patient resources, improved patient engagement in their own care. Increased patient activation and knowledge leads to acceptance of new evidence-based practices and increased quality of care Justification: Local resources help practices and patients improve cardiovascular preventive care and self-management. Patient feedback and involvement in QI promotes patient-clinician communication, tailored health messaging, and patient adherence					
No. co-ops	Name it		Specify it		
	ERIC cluster	ERIC strategy*	Actor	Specific action(s)	Target
5	New	*Engage community resources*	PF, extension agent	Build links between practices and health resources or organizations embedded in communities; varies from meetings with community organizations and participating in collaborative project to participating in referral programs	Practice
1	Support clinicians	Develop resource sharing agreements	Cooperative leadership	Connect to state and county departments of health; connect practices to local health related organizations to participate in collaborative projects	Practice
1	Engage consumers	Involve patients/consumers and family members	Cooperative leadership, PF	Include patients on QI teams	Practice
2	Use evaluative and iterative strategies	Obtain and use patients/consumers and family feedback	Cooperative leadership, PF	Assess and use results of patient/consumers and family feedback from patient engagement surveys; patient focus group, patient and family advisory councils	Practice
1	Engage consumers	Prepare patients/consumers to be active participants	PF	Invite members of the community to participate in tailoring ABCS and CVD messaging for their communities	Practice

*ABCS* aspirin, blood pressure, cholesterol, and smoking cessation; *PF* practice facilitator; *HIT* health information technology; *HIT-PF* health information technology specialist practice facilitator; *EHR* electronic health record; *data expert* health informatics technology expert; *expert consultant* physician faculty or clinical expert; *Q & A* question and answer; *FAQ* frequently asked questions; *extension agent* community-based agent specializing in using community resources to address social determinants of health

*Modifications to ERIC labels and new strategies are in italics. Strategy definitions are in Table 1

### Build health information technology to support data-informed QI

Multiple implementation strategies are needed to achieve data-informed QI. A critical aspect of the cooperatives’ efforts to rapidly disseminate and implement evidence into practice was helping practices gain access to data to support data-informed QI functions (see Table 2). All cooperatives included an *audit and provide feedback* strategy to support data-informed QI. This strategy relied on region- and practice-level HIT infrastructure to deliver trusted data to practices at regular intervals throughout intervention. Audit functions (e.g., ability to produce ABCS performance reports) were necessary for feedback functions (e.g., communication of the audit to clinicians and staff), which in turn, were necessary to inform QI efforts. For instance, generated data were used to identify quality gaps and help to identify opportunities on which to focus improvement efforts. These data were also used to monitor the impact that changes had on ABCS outcomes. *Audit and provide feedback*, thus, was a key strategy to improve ABCS outcomes.

On the ground, up to seven additional ERIC implementation strategies were necessary to accomplish building the infrastructure and capacity needed to implement and sustain *audit and provide feedback* functions, as shown in Table 2. All but one cooperative sought to deliver *audit and provide feedback* functions in a way that could be sustained past the time of their funded project period. The degree to which these seven additional strategies were used by the cooperatives was based on the robustness of existing HIT infrastructure in their region. For example, one cooperative used all seven strategies listed in Table 2, which required significant time and investment. This cooperative hired HIT experts to connect practices’ electronic health record (EHR) systems with external registries (*use data warehousing techniques*) and to develop dashboards—available through centralized portals (*develop and organize quality monitoring systems*)—to provide practices with quality reports needed to support *audit and provide feedback* functions. At times, HIT experts (or an HIT-trained facilitator) worked with practices to change how the practice members documented information from appointments and other sources within their EHR to ensure valid and complete data were extracted (*change records systems*). This step was necessary to get good quality data to a centralized data warehouse, and for the warehouse then to provide the application and cleaned data to support quality reports, which were provided back to practices. This was necessary because many practices did not have the capability to generate robust and useable reports within their own setting. The *local technical assistance* HIT experts provided were extended to assisting practices with translating information from reports to actionable opportunities for improvement related to the generated performance reports and monitoring of progress over time.

### Build QI capacity and improve outcomes

Practice facilitators used a wide range of implementation strategies to build QI capacity and improve outcomes (see Table 3). Facilitation was the central implementation strategy used by all cooperatives. Practice facilitators, who were actors in this process, used many different implementation strategies identified by ERIC to promote practice change and to improve capacity for conducting regular QI to improve ABCS outcomes. For example, facilitators assisted practices in engaging clinicians, practice members, and leaders in practice change efforts (*identify and prepare champions*); organized and facilitated meetings (*organize implementation team and team meetings*); assessed and offered suggestions for revising clinical work flows (*assess and redesign workflow*); assessed HIT needs and data set-up (*assess for readiness and identify barriers and facilitators*); discussed and identified an improvement plan (*develop an implementation blueprint*); assisted with the change process and supported the change efforts including pre-visit planning and outreach (*implementation facilitation*); and helped revise plans based upon results of change cycles. As reported above, practice facilitators were also involved in helping set up HIT infrastructure.

### Enhance clinician and practice member knowledge

Several implementation strategies were employed to enhance clinician and practice member knowledge of evidence-based guidelines and interventions designed to improve ABCS outcomes, measures to assess outcomes, and QI methods (see Table 4). All seven cooperatives included education implementation strategies (e.g., *develop educational materials*, *distribute educational materials*). Cooperatives employed a range of education strategies and more than one strategy might be used by a cooperative. These also included implementation strategies aimed at strengthening peer exchange of best practices to build stronger, sustained supportive professional learning communities. In addition, we identified one new implementation strategy used by EvidenceNOW cooperatives—*create online learning communities*. Given the great distances some cooperatives covered to reach practices, online learning techniques were tested to improve connections between practices for distance learning (e.g., online portals with informational resources, discussion boards, webinars).

### Build community connections and patient involvement

Multiple implementation strategies were used to build community connections and increase patient involvement in improving ABCS outcomes (see Table 5). Some cooperatives used implementation strategies to create or expand community connections with the goal of better connecting patients, practices, and community resources to improve ABCS. For example, one cooperative *developed resource sharing agreements* to forge links between practices and state and county health departments. Five cooperatives worked to *engage community resources* to build connections between practices and health related organizations in the communities, including participation in collaborative projects; this approach is not listed among the ERIC strategies and is newly proposed*.* Two cooperatives employed additional strategies to reach out directly to patients and/or families to promote acceptance of ABCS evidence-based treatments among their patients.

## Discussion

We used the naming and definitions provided by the ERIC taxonomy [10] of implementation strategies to harmonize the language across the EvidenceNOW cooperatives. We expanded on this information by using the descriptive domains recommended by Proctor and colleagues to specify details about how each implementation strategy was actually used. The combination of standardized language from ERIC and specification of what happened when, by whom, under what conditions and for what reason provided a means by which to harmonize, understand, and compare implementation strategies across cooperatives. This is a necessary foundation to cross-cooperative comparisons in a national evaluation such as ESCALATES. Cooperatives, collectively, employed 33 of the ERIC implementation strategies; we identified a number of areas where improvements could be made to the ERIC taxonomy to better align with how implementation happened on-the-ground within the large-scale EvidenceNOW initiative. In addition, we recommend an alternative way of grouping some strategies to better align them with how they are linked to accomplish real-world goals, for example, the need for several HIT-related strategies (e.g., *use data experts*) to implement and sustain use of *audit and provide feedback*.

Cooperatives documented 266 actions mapped to 33 of 73 total ERIC strategies. This number of strategies is consistent with other published studies that have reported a range of 16–59 strategies used in implementation studies [13, 15, 16]. Likely, the range and number of strategies employed in an implementation intervention depends upon the goals and scale of the project. We propose adding three strategies to the ERIC list: *engage community resources*, *create online learning communities*, and *assess and redesign workflow*. Each of these strategies encapsulates distinct actions that were not identified in the existing ERIC taxonomy and are likely to be used in a broad range of other settings. Other researchers have also recommended new strategies: o*btain worker feedback about implementation plan* and *plan for outcome evaluation* [13]. Thus, as ERIC is applied in real world settings, additional strategies may continue to be identified expanding the ERIC taxonomy.

*Audit and provide feedback* was a critical implementation strategy used across cooperatives. *Audit and provide feedback* consists of two distinct yet interrelated concepts: audit, the assessment of performance often compared to a specific target or benchmark; and feedback, communication of the performance. Data used to measure and assess performance must be accurate and credible [23–25]. Many of the individual practices within the EvidenceNOW cooperatives had challenges producing the audit needed for feedback*.* Published trials typically only describe the feedback function within *audit and provide feedback* when reporting findings. In a commentary on an updated Cochrane review of *audit and provide feedback*, the authors list eight characteristics that contribute to variability in effectiveness across studies; all eight characteristics pertain to feedback with no mention of the role and quality of the audit component [24]. Other publications have mentioned audit functions but do not articulate the importance of reliable, sustainable processes to obtain the audits [26] or provide guidance on the infrastructure needed to obtain valid and meaningful audits [25]. Without valid and reliable audits, it is challenging, if not impossible, to provide the feedback, ultimately impacting the capability to reliably identify and monitor progress of QI efforts. Furthermore, pay-for-performance initiatives, such as the Centers for Medicare and Medicaid Quality Payment Program necessitate accurate reporting of clinical quality measures. Thus, there is increasing pressure on primary care practices to generate reliable data in a sustainable manner.

The EvidenceNOW cooperatives described seven strategies that were needed in differing combinations, depending on the local context, to build the necessary HIT infrastructure to support *audit and provide feedback* and to then engage in data-informed QI to improve ABCS outcomes. In the ERIC taxonomy, these seven strategies are spread across four different clusters. We group these HIT strategies based on their shared justification and expected outcomes as this better reflects how these strategies may need to be sequenced to accomplish the intended outcome. Five of the seven strategies to build HIT infrastructure were rated as low-feasibility within the ERIC taxonomy [11] largely because of the difficulty and cost of building HIT infrastructure. Since all but one of the cooperatives planned to sustain *audit and provide feedback* functionality beyond this initiative and because of its focus and substantial funding, EvidenceNOW makes a unique contribution to the ERIC taxonomy by providing insight into what is needed to build the infrastructure to support *audit and provide feedback* functions.

A second clear grouping of strategies is those aimed at helping practices build QI capacity and improve ABCS outcomes, supported foundationally by *implementation facilitation*, the core strategy described by all seven cooperatives. *Implementation facilitation* was used to support practices in accomplishing their QI goals on the path to improving ABCS outcomes; it involves performing interrelated and complex roles and skillfully applying diverse strategies in a flexible and dynamic manner to meet the local needs and priorities of each primary care practice [27–31]. Thus, it is both a role (facilitator) and a strategy (*implementation facilitation*). In EvidenceNOW, practice facilitators provided nearly all of the external support to practices. The facilitators, including facilitators with HIT skill, were responsible for performing, either solely or in conjunction with other actors, 27 of the 33 strategies, spanning all four overarching functions within EvidenceNOW. Using this broad array of strategies requires diverse and sophisticated skills including interpersonal (including emotional intelligence), technical (including adept use of EHR platforms), organizational, communication, leadership, and pedagogical skills [29, 32, 33]. The broad scope of strategies used by facilitators highlights the challenge and complexity of facilitation and the critical role facilitators play in supporting practices in implementing evidence into practice and building capacity for QI.

Regrouping the ERIC strategies from the current nine clusters to a clustering that is more pragmatic may make the ERIC taxonomy easier for practitioners to apply and might be helpful in implementation planning. We intentionally developed these groupings to better reflect how strategies were employed across the diverse contexts of EvidenceNOW. Leeman and colleagues have also proposed an alternative organizing structure for ERIC, proposing five groups, based upon actor and target for each strategy [34]. They suggest that this classification approach, grounded in relevant theory, may aid in understanding the mechanisms by which strategies bring about change [34]. We suggest that the pragmatic grouping we developed may be useful to implementers and recognize the value that analysis of the practical applications of ERIC can have for informing the refinement of this taxonomy for different audiences and users.

In our analysis, we specified further each cooperative’s implementation strategies following Proctor and colleagues’ recommendations. Specifying actor highlighted the broad array of strategies performed by the practice facilitators. A priori specification of dose and temporality, however, was less useful for EvidenceNOW. Most strategies were designed to be used “as needed” in response to local needs of practices. On the one hand, this was a unifying theme across the cooperatives, and on the other hand, specifying these dimensions for each individual strategy was unnecessary. However, the cooperatives did track the activities of their facilitators at varying levels of detail; analysis of this data is outside the scope of this current work. Other researchers have highlighted the challenges with specifying dose: it is resource-intensive to specify and track often because of its ambiguity [12, 15]. However, a priori specification of temporality can be useful in guiding the timing for specific implementation strategies within a more structured multi-site implementation, as highlighted by Huynh and colleagues [15].

### Limitations

Data were collected early in the cooperatives’ implementation phase; they reflected strategies that were included or just started to be employed during the implementation phase of each of the cooperatives’ respective large-scale multi-component interventions. Some cooperatives may have under-reported their strategies because they may have been tacit or intuitive and thus not explicitly reported. This limitation was mitigated through online diary postings by cooperative team members including practice facilitators and early site visits where we observed practice facilitators and others who were delivering implementation strategies, rather than relying exclusively on cooperatives’ self-reported data. The cooperatives did track dose and temporality of strategies used (i.e., when use of a strategy started and ended), but these data were nearly impossible to harmonize across the cooperatives because of the varying level of detail and inconsistencies within how dose and temporality were tracked. It is important to note that this work includes strategies that were specified by cooperatives to provide external support to primary care practices; it does not include micro-level strategies used within individual practices.

## Conclusions

Based on empirical experiences across seven large cooperatives participating in the AHRQ-funded large-scale EvidenceNOW initiative, we recommend refinements to the ERIC list of strategies, which if adopted, would better align ERIC with real-world implementation needs and improve its utility for implementers and researchers. Combined use of the refined ERIC taxonomy and the specifications suggested by Proctor et al. add transparency and thus promote replicability by identifying, creating common nomenclature, and harmonizing a potentially diverse array of implementation strategies used by multiple large multi-site dissemination and implementation initiatives such as EvidenceNOW.

## Additional files

Additional file 1: Example from a single cooperative’s table of interventions. (DOCX 23 kb)

Additional file 2: ERIC strategy names and definitions. (DOCX 52 kb)

## Abbreviations

ABCS: Aspirin use for high-risk patients, blood pressure control, cholesterol management, smoking cessation counseling
AHRQ: Agency for Healthcare Research and Quality
EHR: Electronic health record
ERIC: Expert Recommendations for Implementing Change
ESCALATES: Evaluating System Change to Advance Learning and Take Evidence to Scale, a national evaluation of EvidenceNOW
HIT: Health information technology
QI: Quality improvement
