# Supplementary material for: Specifying and comparing implementation strategies across seven large implementation interventions: a practical application of theory
Source: Implement Sci. 2019 Mar 21;14:32. doi: 10.1186/s13012-019-0876-4 (PMC6429753; doi:10.1186/s13012-019-0876-4)
Supplement: Supplementary file 1 — Example from a single cooperative’s table of interventions. (DOCX 23 kb) [file 13012_2019_876_MOESM1_ESM.docx]

**Additional File 1**

**Example from a Single Cooperative’s Table of Interventions**

The ESCALATES team compiled information from Cooperatives’ proposals and other sources that documented their early activities as described in the Methods section of the main article. The team constructed tables that included a row for each of the Proctor recommended dimensions: Actor, Action, Action Targets, Temporality, Dose, Expected Outcome, and Justification. Tables were member-checked with each respective Cooperative. The following table is an example from one Cooperative’s documented set of activities. “The Action” row lists multiple specific activities; the team mapped each individual activity to an ERIC strategy.

|  | **High-level strategy documented by Cooperative** | | | | | |
| --- | --- | --- | --- | --- | --- | --- |
| **Reporting Dimensions** | **Facilitation** | **EHR/ Data Experts** | **Audit and Feedback** | **Data infrastructure** | **Expert Consultation** | **Community Engagement** |
| Definition | Process of interactive problem solving and support | Expertise to help extract ABCS quality reports | Clinical performance data provided to motivate quality improvement (QI) | Structures that integrate clinical records across facilities and organizations | Experts in change strategies and ABCS guidelines that support implementation | Involving existing community organizations |
| The Actor(s) | Practice Facilitators | Data Experts | Practice Facilitator and Data Experts | Data Experts | Academic Detailers are typically physicians | Practice Facilitator |
| The Action(s) | Assess workflow and patient satisfaction  Assist with improving documentation of ABCS  Audit charts for report validation  Engage leadership  Assist with change process, including PDSAs, educational materials, clinician reminders  Connect practices with community organizations | Help practices run ABCS reports;  Help Practice Facilitators understand how to interpret and validate measures | Audit report of ABCS data is shared with practices  Discuss and identify improvement plan  Monitor improvement over time | informatics experts extract and connect data from practice EHR to data warehouse/data exchange  Data experts negotiate with EHR vendors  Data Experts help validate data with practice | Education and outreach directly to practices on clinical topics  Education and support for Practice Facilitators | Link practices and resources in their community  Provide information to practices about community resources and activities  Potentially partner with practices on ABCS improvement |
| Action Targets | Practice | Practice; Practice Facilitators | Clinician and clinical team | Practice; other relevant healthcare organizations in the region | Clinicians and Practice Facilitators | Practice |
| Temporality | 12-month intervention;  28 visits by Practice Facilitator | Mobilized at beginning of intervention, and as needed  Quarterly data reports pulled for practice | Quarterly reports | Once connected always available | Practice gets visit once every six months, at start of new intervention phase  Practice Facilitator exposure to consultant, by request | CHIOs hold monthly meetings |
| Dose | Visit length and quality vary, ranging from 30-minutes to half-day; some visits used for research data collection | Data experts work with practices as much as needed until data are extracted | Quarterly  Reports reviewed during visit or quality improvement meeting | Data experts work with practices as much as needed until practice connected to HIE; access to HIE constant | Visits are approximately 1-1.5 hours | Board members assemble for 1-4 hours monthly; usual meetings are 1 hour |
| Implementation outcome affected | ABCS quality measures  Healthy Community / Connect with CHIO  (if applicable)  Financial Security  Joy in Practice | Heath IT capacity  Ability to generate ABCS reports  Documentation of ABCS | Documentation of ABCS  Delivery of ABCS | Health IT capacity  Data interoperability / sharing  Documentation of ABCS  Reporting capacity | Ensure knowledge of evidence-based guidelines | Use of local resources by practices and patients to improve ABCS |
| Justification | Practices are under-resourced for quality improvement; Practice Facilitators serve as resource for QI | Practices have little or no IT capacity; Data Experts serve as a resource to practice | Practice need to see their own data to motivate them to improve | Practices need population level data to improve quality  Practices need interoperability to optimize care delivery | Clinicians listen to other clinical experts; guidelines can change rapidly and Expert Consultants address knowledge deficits | Practices do not know about all the resources available; rural communities lack resources that working together can help them obtain |
