# Supplementary material for: Specifying and comparing implementation strategies across seven large implementation interventions: a practical application of theory
Source: Implement Sci. 2019 Mar 21;14:32. doi: 10.1186/s13012-019-0876-4 (PMC6429753; doi:10.1186/s13012-019-0876-4)
Supplement: Supplementary file 2 — ERIC strategy names and definitions. (DOCX 52 kb) [file 13012_2019_876_MOESM2_ESM.docx]

**Additional File 2
ERIC Strategy Names and Definitions**

Below are the 33 ERIC implementation strategies to which we mapped cooperative activities, plus the three new strategies we named and defined. Broadening the definition and/or the name of particular ERIC strategies enabled us to map cooperative intervention activities to these ERIC strategies and retain the character of these activities. We listed strategies in the order they appear in Table 1. Any changes we made to existing names and definitions, including additions or substantial deletions, plus the new names and definitions we created, are marked in *italics.*

| **ERIC Strategy^[[1]](#endnote-1)^ or**  **New Label** | **ERIC Strategy Definition and Ancillary Material^[[2]](#endnote-2)^** | **ERIC Strategy List Number1** | | **ERIC Cluster1** |
| --- | --- | --- | --- | --- |
| 1. Use Data Experts | Involve, hire, and/or consult experts to *acquire, structure, manage, report, and use* data generated by implementation efforts.  Ancillary Material:  *It is sometimes necessary to engage* data experts early in the implementation planning process. *Data Experts* *may be particularly important to enable effective use of data-driven clinical innovations and to support other strategies such as: Develop Implementation Tools for Quality Monitoring, Develop and Organize Quality Monitoring Systems, Audit and Provide Feedback, Change Record Systems, Use Data Warehousing Techniques.* | 67 | | Adapt and tailor to context |
| 1. Use Data Warehousing Techniques | Integrate clinical records across facilities and organizations to facilitate implementation across systems.  Ancillary Material:  *A key feature of this strategy is linking local data to data platforms outside the local setting. Often the connection is to a central data repository that provides benchmarking capability (see Audit and Provide Feedback) or other aggregation functions.* Records that include variables that can serve as outcome measures are particularly useful. When outcomes of interest are not available, it may be useful to examine proxy measures. | 68 | | Adapt and tailor to context |
| 1. Develop and Implement Tools for Quality Monitoring | Develop, test, and introduce into quality-monitoring systems the right input – the appropriate language, protocols, algorithms, standards, and measures (of processes, patient/consumer outcomes, and implementation outcomes) that are often specific to the innovation being implemented.  Ancillary Material:  These tools should be flexible enough to reflect fidelity, even after adaptations to the setting or client. Performance sites can benefit when these tools are available locally, particularly to help clinicians develop a sense of ownership for the change process. Quality monitoring tools can be coordinated with other strategies to encourage or reward performance that is in alignment with the clinical innovation. See Krein et al.^[[3]](#endnote-3)^ for an example of this process. *This strategy may include activities necessary to prepare data needed to support the Develop and Organize Quality Monitoring Systems and the Audit and Provide Feedback strategies, including data cleaning, validation, and standardization.* | 26 | | Use evaluative and iterative strategies |
| 1. Develop and Organize Quality Monitoring Systems | Develop and organize systems and procedures that monitor clinical processes and/or outcomes for the purpose of quality assurance and improvement.  Ancillary Material:  This includes developing systems for monitoring through peer reviews, collecting data from patients and consumers, clinicians, and supervisors, and using administrative and electronic record data. This category of strategies also includes the design of disease-specific clinical registries *and dashboards*, where clinical information and tools (graphical representations, real-time report cards, comparisons with benchmarks, *fidelity* *monitoring*, etc.) are *easily* available to *key stakeholders and users, e.g.,* care team members. These systems may *provide* *support to the Audit and Provide Feedback strategy*. Some intensive fidelity monitoring activities (e.g., psychotherapy recordings) are more practical at random, but not infrequent, intervals. | 27 | | Use evaluative and iterative strategies |
| 1. Fund and Contract *(and/or Negotiate) with Vendors* for the Clinical Innovation | Governments and other payers of services issue requests for proposals to deliver the innovation, use contracting processes to motivate providers to deliver the clinical innovation, and develop new funding formulas that make it more likely that providers will deliver the innovation.  Ancillary Material:  None | 34 | | Utilize financial strategies |
| 1. Change Record Systems | Change clinical documentation (e.g., electronic medical records) systems to allow better assessment of implementation or clinical outcomes.  Ancillary Material:  *This strategy involves changing or upgrading the structure, content, function, or design of record system components. These systems most commonly involve the electronic health or medical records systems (EHR/EMR).* These changes may include modifying the format of progress notes and treatment plans to reflect the innovation (evidence-based practice) being implemented. *This strategy focuses on changes within the clinical setting. Please refer to Use* *Data Warehousing Techniques for activities that involve links to or integration with outside entities, repositories, or systems.* | 12 | | Change infrastructure |
| 1. Provide Local Technical Assistance | Develop and use a system to deliver technical assistance *within local settings that is* focused on implementation issues.  Ancillary Material:  Local technical assistants can be *local staff members or affiliated with a* broader or centralized network of technical assistants. *The key is providing tailored technical assistance within settings where the clinical innovation is being implemented.* Technical assistance for both the clinical innovation and the implementation processes may be important. For example, the VA aims to have mental health Evidence-Based Psychotherapy coordinators, Military Sexual Trauma coordinators, and OEF/OIF/OND coordinators in each facility who can provide technical assistance to other local clinicians for relevant initiatives. *This strategy may be supported by Centralize Technical Assistance, which may provide necessary infrastructure and support for Provide Local Technical Assistance.* | 54 | | Provide interactive assistance |
| 1. Audit and Provide Feedback | *Develop summaries of* clinical performance over a specific time period*, often including a comparator,* and give it to clinicians and*/or* *administrators. Summary content (e.g., nature of the data, choice of comparator) and their delivery (e.g., mode, format) are designed* to modify *specifically targeted behavior(s) or actions of individual practitioners, teams, or health care organizations*.^[[4]](#endnote-4)^  Ancillary Material:  *Brehaut and colleagues^[[5]](#endnote-5)^* *list 15 suggestions for optimal outcomes using this strategy in clinical settings.* *Feedback* may be *derived* from a variety of sources, including *Quality Monitoring Systems*, medical records, computerized databases, observation, or feedback from patients. Performance evaluations may also be considered as audit and feedback data if the evaluation included specific information on clinical performance. Feedback summaries may include recommendations. Feedback may be displayed publicly, and often involves comparisons to peers or to local, state, national, or international norms *or benchmarks*. Feedback may be designed to guide a clinician in improving fidelity. It should also be noted that audit and feedback data can be helpful in promoting the continuation of intended behavior. Performance data may include process variables, outcomes, or fidelity measures. Feedback can include mandatory performance measures, which are related to benchmarks from the literature or normative data within an organization or industry. *This strategy may rely on systems described by the Develop and Organize Quality Monitoring Systems strategy. Audit and Provide Feedback includes pre-defined summaries of data that are specifically designed to trigger change in behavior to improve clinical performance. Feedback is structured and provided at regular pre-defined intervals. These features differentiate this strategy from Develop and Organize Quality Monitoring Systems, which is focused on continuous monitoring via, e.g., dashboards or registries.* | 5 | | Use evaluative and iterative strategies |
| 1. Use an Implementation Advisor | Seek guidance from experts in implementation, *including providing support and training for the implementation work force*  Ancillary Material:  This could include consultation with outside experts such as university-affiliated faculty members or hiring quality improvement experts or implementation professionals*. Implementation Advisors may provide support and training for facilitators (see Implementation Facilitation) related to broad facilitation skills, how to use and interpret data, effective use of an EHR.* | 65 | | Develop stakeholder inter-relationships |
| 1. *Implementation* Facilitation | *“[A] multi-faceted process of enabling and supporting individuals, groups and organizations in their efforts to adopt and incorporate clinical innovations into routine practices,”^[[6]](#endnote-6)^*  Ancillary Material:  *Clinical innovations that are the target of facilitation efforts can include a continuum of innovations ranging from evidence-based research evidence to incremental quality improvement efforts. Ideally, facilitation efforts are holistic and help to build capacity and skill within the setting to sustain ongoing clinical innovation/improvement*. Facilitation can be *provided by individuals who are* internal or external *to the setting within which Implementation Facilitation occurs*. *Implementation Facilitation is* *an* interactive support process *and draws on* a combination of implementation strategies *based on the needs of the setting*. *Implementation Facilitation is sometimes referred to as a “meta” strategy because*, as an interactive support process, it often includes a combination of implementation strategies and bundles multiple strategies as needed. | 33 | | Provide interactive assistance |
| 1. Assess for Readiness and Identify Barriers and Facilitators | Assess various aspects of an organization to determine its degree of readiness to implement *and identify* barriers that may impede implementation and strengths that can be *leveraged to facilitate* the implementation effort.  Ancillary Material:  Readiness assessments may focus on agency finances, staffing levels, and other material or logistical resources needed, or available, to support the implementation effort. Further this assessment may also focus on leadership support, the organizational priority for change, and the presence of successful experience with quality improvement techniques and change management. Additional aspects for assessment may include other services provided, as well as community support, stakeholder attitudes, and beliefs and perceptions of evidence for the innovation or change. Rationale for current practices, organizational climate and culture, structure, decision-making styles, and the perceived needs of frontline stakeholders to implement the change or innovation (consider adaptation needs and limits) are also important aspects to consider in this assessment. Readiness assessments can be used to vet, eliminate, or prioritize implementation sites. More so, the assessment can help make internal decisions about whether to go ahead with an implementation initiative. Some barriers can be difficult to observe prior to implementation. Specific measures have been created to assess readiness for change, which may be useful.^[[7]](#endnote-7),^ ^[[8]](#endnote-8),^^[[9]](#endnote-9)^ *This strategy does not include Assess and Redesign* *Workflow*. | 4 | | Use evaluative and iterative strategies |
| 1. Develop *an* Implementation Blueprint | Develop a formal implementation blueprint that includes all goals and strategies. The blueprint should include the following: 1) aim/purpose of the implementation; 2) scope of the change (e.g., what organizational units are affected); 3) timeframe and milestones; and 4) appropriate performance/progress measures. *Use and update this plan to guide the implementation effort over time*.  Ancillary Material:  The implementation blueprint or manual may be informed by one or more theories or conceptual frameworks and/or data from pre-implementation needs assessments. This blueprint can also provide a useful historical record of the implementation process, as well as provide a mechanism to track changes over time. The implementation blueprint is often useful to ensure feedback is received from prospective frontline users of the blueprint prior to implementation.  Consider coordinating this strategy with the development of a fidelity monitoring tool.  Issues to consider separately, especially for research purposes:  • Number and type of implementation strategies  • Organizational levels involved—this can vary by type of intervention. It may be possible to do some interventions at the lowest level. Others may require top management.  • Pre-implementation assessments would be separate step  Other examples of how to create an implementation blueprint include the CDC's Replicating Effective Programs.^[[10]](#endnote-10),^^[[11]](#endnote-11)^  Examples of projects using a blueprint include:  • HI-TIDES^[[12]](#endnote-12)^  • Depression in a substance abusing population^[[13]](#endnote-13)^  • Mental health services in federally qualified health centers^[[14]](#endnote-14)^ | 23 | | Use evaluative and iterative strategies |
| 1. Conduct Cyclical Small Tests of Change | Implement changes in a cyclical fashion using small tests of change before taking changes system-wide. Tests of change benefit from systematic measurement, and results of the tests of change are studied for insights on how to do better. This process continues serially over time, and refinement is added with each cycle.  Ancillary Material:  Two common small tests of change cycling strategies are “Plan-Do-Study-Act” from Deming’s quality management work,^[[15]](#endnote-15)^ and six sigma’s Define- Measure- Analyze-Improve-Control sequence.^[[16]](#endnote-16)^ | 14 | | Use evaluative and iterative strategies |
| 1. *Assess and Redesign Workflow* | *Observe and map current work processes and plan for desired work processes, identifying changes necessary to accommodate, encourage, or incentivize use of the clinical innovation as designed.^[[17]](#endnote-17)^*  *Ancillary Material:*  *Workflow redesign^[[18]](#endnote-18)^ may comprise consideration of the following:*   - *Perceived process (what we think is happening);* - *Reality process (what the process actually is); and* - *Ideal process (what the process could be).*   *Process mapping is a core technique for system change. Process maps are used to develop plans that may be a part of Develop an Implementation Blueprint.* | -- | | -- |
| 1. Remind Clinicians | Develop reminder systems designed to help clinicians to recall information and/or prompt them to use the clinical innovation.  Ancillary Material:  Reminders could be patient or encounter-specific, provided verbally, on paper, or electronically. Computer-aided decision support and drug dosages are included in this strategy. Reminders may be delivered at various time points (prior to service, during service, or following service delivery). | 58 | | Support clinicians |
| 1. Identify and Prepare Champions | Identify and prepare individuals who dedicate themselves to supporting, marketing, and driving through an implementation, overcoming indifference or resistance that the intervention may provoke in an organization.  Ancillary Material:  This strategy includes preparing individuals for their role as champions. Champions are primarily internal to the organization. Additional issues raised include the need for guidance regarding:   1. Methods and considerations related to the selection and identification of champions. Social network theory and methods may be useful in this regard. 2. Training and or providing champions support materials. 3. Addressing incentives or disincentives to the champion role. 4. Whether there are needs for champions at different levels of an organization (e.g., clinic, region, national).   Champions are often distinguished from opinion leaders. Opinion leaders may be considered more of an objective third party with relevant expertise. | 35 | | Develop stakeholder inter-relationships |
| 1. Recruit, Designate, and Train for Leadership | Recruit, designate, and train leaders for the change effort.  Ancillary Material:  Change efforts require certain types of leaders, and organizations may need to recruit accordingly, rather than assuming that their current personnel can implement the change. Designated change leaders can include an executive sponsor and a day-to-day manager of the effort. Change leaders should consider how to establish effective supervisory lines for clinical practice innovations that are enacted by clinicians when the change leader does not have similar clinical responsibilities. | 57 | | Develop stakeholder inter-relationships |
| 1. Organize *Implementation Teams and Team Meetings* | Develop and support teams of clinicians*, staff, patients and other stakeholders* who are implementing *or may be users of* the innovation. *Provide* protected time *for teams* to reflect on the implementation *progress*, share lessons learned, *make refinements to plans,* and support one another’s learning.  Ancillary Material:  None | 48 | | Develop stakeholder inter-relationships |
| 1. Intervene with Patients/Consumers to Enhance Uptake and Adherence | Develop strategies with patients to encourage and problem solve around adherence.  Ancillary Material:  This includes patient/consumer reminders*, outreach,* and financial incentives to attend appointments. Feedback regarding patient/consumers' understanding and use of the treatment is also important to collect. | 39 | | Engage consumers |
| 1. Develop Educational Materials | Develop and format manuals, toolkits, and other supporting materials *to* make it easier for stakeholders to learn about the innovation and for clinicians to learn how to deliver the clinical innovation*. This can include technology-delivered (e.g., online/smartphone-based static or dynamic) content and health messaging.*  Ancillary Material:  Create eye-catching, easy-to-use educational documents. Distill complex information into easier-to-learn components. Consider teaching skills modularly. Use different forms of media, and target messages for different audiences. Educational materials should reflect principles of adult learning theory. Assessment of current, available technology infrastructure to accommodate educational media (e.g., firewalls, old hardware, old software) is merited. Consider how the educational materials will be used over time. For example, will the educational materials’ primary use be to train new or rotating staff; or to refresh staff knowledge; or to be incorporated into existing supervision, competency, and performance review structures. Educational materials may be refined through the use of formative evaluation feedback.  Relevant suggestions are provided via the REP framework, under its ‘packaging’ domain.^[[19]](#endnote-19)^ Further support related to developing educational materials can be found on the Training Within Industry Service website: <http://twi-institute.org/training-within-industry> | 29 | | Train and educate stakeholders |
| 1. Distribute Educational Materials | Distribute educational materials (including guidelines, manuals and toolkits) in person, by mail, and/or electronically.  Ancillary Material:  None | 31 | | Train and educate stakeholders |
| 1. Conduct Educational Outreach Visits | Have a trained person meet with *individuals or teams* in their *work* settings to educate *them* about the clinical innovation with the intent of changing *behavior to reliably use the clinical innovation as designed*.  Ancillary Material:  Visits to the site may be in-person or virtual, via the Internet. Some initiatives may require regular educational outreach as part of maintaining *behavioral changes required to ensure sustained, consistent use of the innovation*. Academic detailing is another commonly used term, although academic detailing typically involves many additional discrete implementation strategies (e.g., conduct ongoing training, modeling, developing and distributing educational materials^[[20]](#endnote-20),^^[[21]](#endnote-21)^). | 16 | | Train and educate stakeholders |
| 1. Provide Ongoing Consultation | Provide ongoing consultation with one or more experts in the strategies used to support implementing the innovation.  Ancillary Material:  Ongoing consultations could include in-person or distance consultation and feedback on taped clinical encounters. Consultations are tailored to the clinician’s actual practice, thus, differentiating a consultation from ongoing trainings. Feedback may be from a consultant external to the organization, which distinguishes consultation from clinical supervision. Some practice changes can involve a recertification process, thus, involving consultation ensures adequate fidelity. Consultation may also be necessary for non-clinical staff such as administrators and those responsible for billing, constructing feedback systems, or other staff with duties that impact the implementation process. | 55 | | Train and educate stakeholders |
| 1. Conduct Ongoing Training | Plan for and conduct training in the clinical innovation in an ongoing way *for all individuals involved with implementation and users of the clinical innovation e.g., clinicians, implementation staff, practice facilitators.*  Ancillary Material:  This can include follow-up training, advanced training, booster training, purposefully spaced training, training to competence, integration of off the- job and on-the-job training, structured supervision, the introduction of concepts in a specific sequence to ensure mastery, and trainings based on the level of clinician knowledge. Ongoing training efforts need to reach across shifts and accommodate staff turnover, as well as rotating staff (e.g., residents). Trainings can be in-person, on the web, or technology-assisted (e.g., simulation lab training), and may focus on individuals or involve groups. When planning for ongoing training, it is important to describe the training components, including the timing and frequency of trainings. Issues related to the dynamics of training can be found in the strategy, make training dynamic. | 19 | | Train and educate stakeholders |
| 1. Create a Learning Collaborative | Facilitate the formation of groups of providers or provider organizations and foster a collaborative learning environment to improve implementation of the clinical innovation.  Ancillary Material:  There are several approaches to this in the literature including peer consultation networks, online communities of practice, quality circles, and learning collaboratives. Groups may meet in person or interact using a wide variety of media. The inclusion of a quality manager within the collaborative may be useful. Positive deviance approaches use “discovery and action dialogue” among peers to promote collaborative learning.^[[22]](#endnote-22),^^[[23]](#endnote-23),^^[[24]](#endnote-24)^  Resources specific to learning collaboratives include:  *The Agency for Healthcare Research Quality (AHRQ)^[[25]](#endnote-25)^*  The Institute for Healthcare Improvement (IHI)^[[26]](#endnote-26),^^[[27]](#endnote-27)^  Key terms for searching literature specific to collaborative learning include:  learning community, learning network, and community of practice. | 20 | | Train and educate stakeholders |
| 1. *Create Online Learning Communities* | *Create an online portal for clinical staff members to share and access resources, webinars, and FAQs related to the specific evidenced-based intervention, and provide interactive features to encourage learning across settings and teams, e.g., regular blogs, facilitated discussion boards, access to experts, and networking opportunities.*  *Ancillary Material:*  *None* | -- | | -- |
| 1. Conduct Educational Meetings | Hold meetings targeted toward *educating* *multiple* stakeholder groups (i.e. providers, administrator*s*, other organizational stakeholders, community *members*, patient*s*/consumer*s*, famil*ies*) *about the clinical innovation and/or its implementation.*  Ancillary Material:  The content of the education may include information regarding what to expect as implementation moves forward. It *may be* useful to *consider* *whether* meeting attendees are relatively *homogeneous or comprised of multiple roles and disciplines* so that the education can *be most effective in meeting* stakeholder needs. For example, some educational meetings may inform the stakeholder group about the clinical innovation in a way intended to increase demand *by including patients and families,* while others may preview *technical facets of* the clinical innovation for providers and administrators. It is often useful to have recordings or other materials from the educational meetings available to those who cannot attend the meetings *and for individuals engaged or hired after the* meeting*(s).* | 15 | | Train and educate stakeholders |
| 1. Shadow Other Experts | Provide ways for key individuals to directly observe experienced people engage with or use the targeted practice change/innovation.  Ancillary Material:  While shadowing traditionally has involved in-person observation, creative use of technology may provide additional opportunities for individuals to observe and learn from those experienced in the innovation. | 60 | | Train and educate stakeholders |
| 1. Visit Other Sites | Visit sites where a similar implementation effort has been considered successful.  Ancillary Material:  Clarifying the goals of the site visit prior to making the visit is particularly useful. Comparing and contrasting the features of one’s own site with the comparison site in preparation for the visit may better inform the visit objectives. Clarifying goals, in part includes developing a plan for using the information upon returning to your setting. Identify adaptations made in implementing the innovation and any perceived impact on the effectiveness of the innovation/practice change. It is important to document facilitators and lessons learned. Much can be learned from visiting sites that have a strong track record for successfully implementing a wide variety of other innovations/practice changes. Consulting with sites where implementation has stalled or failed can also provide useful information. Sites also benefit from sharing implementation planning and execution notes virtually (i.e., information exchange is not limited to physical visits). | 72 | | Develop stakeholder inter-relationships |
| 1. Promote Network Weaving | Identify and build on existing high quality working relationships and networks within and outside the organization, organizational units, teams, etc. to promote information sharing, collaborative problem-solving, and a shared vision/goal related to implementing the innovation.  Ancillary Material:  Individuals functioning as network weavers usually have external links outside of the community to bring in information and ideas. An example would be nurses and doctors who staff hospitals and skilled nursing facilities, and the patients who rotate among these facilities. Networks are somewhat more organic than collaboratives and are often enduring and durable. See: <http://www.networkweaver.com/> | 52 | | Develop stakeholder inter-relationships |
| 1. Capture and Share Local knowledge | Capture local knowledge from implementation sites on how implementers and clinicians made something work in their setting and then share it with other sites.  Ancillary Material:  This strategy is often coordinated with centralized technical assistance and learning collaboratives. There are multiple techniques for capturing local knowledge, which could be presented in multiple formats. For example, short YouTube videos could be created that document testimonials from clinicians who have successfully used a given innovation. Another example would be maintaining a running list of a team's response to specific implementation barriers that could be shared readily through a platform like GoogleDocs or Microsoft SharePoint. Additional techniques can be found at [www.liberatingstructures.com](http://www.liberatingstructures.com). | 7 | | Develop stakeholder inter-relationships |
| 1. *Engage Community Resources* | *Connect practices and their patients to community resources outside the practice (e.g., state and county health departments; non-profit organizations; resources related to addressing the social determinants of health; and organizations focused on self-management techniques and support).*  *Ancillary Material:*  *None* | -- | | -- |
| 1. Develop Resource Sharing Agreements | Develop partnerships with organizations that have resources needed to implement the innovation.  Ancillary Material:  For example, this could involve data sharing agreements, agreements to share necessary equipment (e.g., telemedicine equipment), or sharing the cost of bringing in experts who provide training and consultation. Resource sharing agreements could involve formal memorandums of understanding (MOUs), or be much more informal in nature. | 30 | | Support clinicians |
| 1. Involve Patients/Consumers and Family Members | Engage or include patients/consumers and families in the implementation effort.  Ancillary Material:  Feedback from stakeholders can be obtained at any stage of the implementation process depending on the needs and goals of project. Involving stakeholders in the pre-implementation phase for many innovations is advantageous. Training in the innovation, and relevant advocacy, may also be included in stakeholder involvement. Informal caregivers such as neighbors, friends, and other key sources of support may also be prudent to include. | 41 | | Engage consumers |
| 1. Obtain and Use Patients/Consumers and Family Feedback | Develop strategies to increase patient/consumer and family feedback on the implementation effort.  Ancillary Material:  This can continue throughout the implementation effort. Strategies could include complaint forms, or methods, which funnel feedback to change managers or advisory boards. Consider whether anonymous feedback formats are appropriate. | 46 | | Use evaluative and iterative strategies |
| 1. Prepare Patients/Consumers to Be Active Participants | Prepare patients/consumers to be active in their care, to ask questions, and specifically to inquire about care guidelines, the evidence behind clinical decisions, or about available evidence-supported treatments.  Ancillary Material:  Preparing consumers to inquire about specific practices can involve asking questions, and educating patients/consumers about the existence of treatments supported by evidence, as well as explicitly inviting them into the process of treatment decision-making. | 50 | | Engage consumers |
| Note: this strategy overlapped with ones that we expanded above. We revised this one accordingly. | | |  |  |
| Centralize Technical Assistance | Develop and use a centralized system to deliver technical assistance focused on implementation issues. *This strategy may involve setting up a Centralized Technical Assistance entity that may, in turn, support a network of technical assistants who work directly with local implementation sites. It may provide human-driven ad hoc support as with a helpdesk, from a centralized location (online or phone) or may be automated e.g., online frequently asked questions (FAQs) or via online community with discussion boards.*  Ancillary Material:  This could be the designation of a lead technical assistance organization (could also be responsible for training). The lead technical assistance entity can develop other mechanisms (e.g., call-in lines or websites) in order to share information on how to best implement the clinical innovation. *This centralized function may support Provide Local Technical Assistance and enable local implementation sites to Use Data Experts.* | 8 | | Provide interactive assistance |

1. **References**

   Waltz TJ, Powell BJ, Matthieu MM, Damschroder LJ, Chinman MJ, Smith JL, et al. Use of concept mapping to characterize relationships among implementation strategies and assess their feasibility and importance: results from the Expert Recommendations for Implementing Change (ERIC) study. *Implementation Science*. 2015;10:109. [↑](#endnote-ref-1)
2. # Powell BJ, Waltz TJ, Chinman NJ, Damschroder LJ, Smith JL, et al. A refined compilation of implementation strategies: results from the Expert Recommendations for Implementing Change (ERIC) project; Additional File 6: Expert Recommendations for Implementing Change (ERIC) – Discrete Implementation Strategy Compilation with Ancillary Material. *Implementation Science*. 2015;10:21.

   [↑](#endnote-ref-2)
3. Krein SL, Bernstein SJ, Fletcher CE, Makki F, Godzweig CL, Watts B, et al. Improving eye care for veterans with diabetes: An example of using the QUERI steps to move from evidence to implementation: QUERI series. *Implementation Science.* 2014, 3:1–11. [↑](#endnote-ref-3)
4. Brehaut JC, Eva KW. Building theories of knowledge translation interventions: use the entire menu of constructs. *Implementation Science*. 2012; 22:7(1):114. [↑](#endnote-ref-4)
5. Brehaut JC, Colquhoun HL, Eva KW, Carroll K, Sales A, Michie S, Ivers N, Grimshaw JM. Practice Feedback Interventions: 15 Suggestions for Optimizing Effectiveness. *Annals of Internal Medicine*. 2016;164(6):435-41. [↑](#endnote-ref-5)
6. Ritchie MJ, Dollar KM, Miller CJ, Oliver KA, Smith JL, Lindsay JA, et al. Using Implementation Facilitation to Improve Care in the Veterans Health Administration (Version 2). Veterans Health Administration, Quality Enhancement Research Initiative (QUERI) for Team-Based Behavioral Health. 2017:1. Available at: <https://www.queri.research.va.gov/tools/implementation/Facilitation-Manual.pdf>. [Last accessed 7/18/18] [↑](#endnote-ref-6)
7. Helfrich CD, Li Y-F, Sharp ND, Sales AE: Organizational readiness to change assessment (ORCA): Development of an instrument based on the promoting action on research in health services (PARIHS) framework. *Implementation Science*. 2009;4:38. [↑](#endnote-ref-7)
8. Lehman WEK, Greener JM, Simpson DD. Assessing organizational readiness for change*. Journal of Substance Abuse Treatment*. 2002, 22:197–209. [↑](#endnote-ref-8)
9. Weiner BJ, Amick H, Lee SY. Conceptualization and measurement of organizational readiness for change: A review of the literature in health services research and other fields. *Medical Care Research and Review.* 2008; 65:379–436. [↑](#endnote-ref-9)
10. Neumann MS, Sogolow ED. Replicating effective programs: HIV/AIDS prevention technology transfer. *AIDS Education and Prevention.* 2000; 12(5 Suppl):35–48. [↑](#endnote-ref-10)
11. Kilbourne AM, Neumann MS, Pincus HA, Bauer MS, Stall R. Implementing evidence-based interventions in health care: Application of the replicating effective programs framework. *Implementation Science*. 2007;2:1–10. [↑](#endnote-ref-11)
12. Pyne JM, Fortney JC, Curran GM, Tripathi S, Atkinson JH, et al. Effectiveness of collaborative care for depression in human immunodeficiency virus clinics. *Archives of Internal Medicine*. 2010; 171:23–31. [↑](#endnote-ref-12)
13. Curran GM, Mukherjee S, Allee E, Owen RR. A process for developing an implementation intervention: QUERI series. *Implementation Science*. 2008;3:1–11. [↑](#endnote-ref-13)
14. Hunt JB, Curran G, Kramer T, Mouden S, Ward-Jones S, et al. Partnership for implementation of evidence-based mental health practices in rural federally qualified health centers: Theory and methods. *Progress in Community Health Partnerships.* 2012;6:389–398. [↑](#endnote-ref-14)
15. Deming WE. *Out of the Crisis*. Cambridge, MA: MIT Press; 1986. [↑](#endnote-ref-15)
16. Linderman K, Schroeder RG, Zaheer S, Choo AS. Six sigma: A goal-theoretic perspective. *Journal of Operations Management.* 2003;21:193–203. [↑](#endnote-ref-16)
17. Strongwater SL and Pelote V. *Clinical Process Redesign: A Facilitator’s Guide*. Silver Spring, MD: Aspen Publications; 1996. [↑](#endnote-ref-17)
18. Module 5. Mapping and Redesigning Workflow. Agency for Healthcare Research and Quality, Rockville, MD. Available from: <http://www.ahrq.gov/professionals/prevention-chronic-care/improve/system/pfhandbook/mod5.html>. Content last reviewed May 2013. [Accessed 7/18/2018] [↑](#endnote-ref-18)
19. Kilbourne AM, Neumann MS, Pincus HA, Bauer MS, Stall R. Implementing evidence-based interventions in health care: Application of the replicating effective programs framework. *Implementation Science*. 2007; 2:1–10. [↑](#endnote-ref-19)
20. Fischer MA, Avorn J. Academic detailing can play a key role in assessing and implementing comparative effectiveness research findings. *Health Affairs* (Millwood). 2012; 31:2206–2212. [↑](#endnote-ref-20)
21. Soumerai SB, Avorn J. Principles of educational outreach (‘academic detailing’) to improve clinical decision making. *JAMA*;1990,4:549–556. [↑](#endnote-ref-21)
22. Positive deviance initiative: Discovery and action dialogue (DAD) cheat sheet. Available from: <https://web.archive.org/web/20160415111014/http://www.positivedeviance.org/pdf/DAD%20cheat%20sheet%202.pdf>. [Accessed 7/18/18] [↑](#endnote-ref-22)
23. Pascale R, Sternin J, Sternin M: *The Power of Positive Deviance: How Unlikely Innovators Solve the World’s Toughest Problems*. Boston, MA: Harvard Business Press; 2010. [↑](#endnote-ref-23)
24. Bradley EH, Curry LA, Ramanadhan S, Rowe L, Nembhard IM, Krumholz HM. Research in action: Using positive deviance to improve quality of health care. *Implementation Science*; 2009, 4:1–11. [↑](#endnote-ref-24)
25. Agency for Healthcare Research and Quality. Establishing an AHRQ Learning Collaborative, A White Paper: Developing an AHRQ Learning Collaborative. Created March 2012. Last reviewed Oct 2014. Available from: <https://archive.ahrq.gov/research/findings/final-reports/learningcollab/learning5.html> [Last Accessed 7/18/18] [↑](#endnote-ref-25)
26. Institute for Healthcare Improvement. Collaboratives. 2018. Available from: <http://www.ihi.org/Engage/collaboratives/Pages/default.aspx>. [Last Accessed 7/18/18] [↑](#endnote-ref-26)
27. Institute for Healthcare Improvement: *The Breakthrough Series: IHI’s Collaborative Model for Achieving Breakthrough Improvement*. Cambridge, Massachusetts: Institute for Healthcare Improvement; 2003. [↑](#endnote-ref-27)
